# Linking the Pre‐Assessment Information Form (PIF) to the ICF: Enhancing Standardized Functional Assessment in Parkinson's Disease

**DOI:** 10.1002/pri.70247

**Published:** 2026-06-13

**Authors:** Fernanda Botta Tarallo Rogatto, Érica Tardelli Neves Guelfi, Vívian Elaine Vargas Alflen, Leandro Bruno Barbosa Silva, Gabriela Santos Pereira, Soraia Micaela Silva

**Affiliations:** ^1^ Department of Physiotherapy Associação Brasil Parkinson São Paulo São Paulo Brazil; ^2^ Postgraduate Program in Rehabilitation Sciences Universidade Nove de Julho (UNINOVE) São Paulo São Paulo Brazil

**Keywords:** international classification of functioning, Parkinson disease, patient outcome assessment

## Abstract

**Background and Purpose:**

Systematic documentation of functioning in people with Parkinson's disease (pwPD) requires a standardized language to enable comparability across services and countries. The International Classification of Functioning, Disability and Health (ICF) provides this framework; however, instruments recommended by the European Physiotherapy Guideline for Parkinson's disease have not previously been linked to its categories. This study aimed to link items from the Pre‐Assessment Information Form (PIF) to ICF categories using the refined linking rules.

**Methods:**

Methodological study. Two independent evaluators with extensive knowledge of the ICF taxonomic framework applied 10 refined linking rules to 26 PIF items, working independently and blindly. Disagreements were resolved by a third evaluator with prior ICF experience. Inter‐rater agreement was assessed using the Kappa coefficient (95% confidence interval).

**Results:**

Initial inter‐rater agreement was moderate across all ICF taxonomy levels (*k* = 0.42–0.54; *p* < 0.001), decreasing as category specificity increased. Following a final consensus, 26 PIF items were linked to 25 unique ICF categories and 34 linked concepts across two components: Activities and Participation (21 categories; 84.0%) and Body Functions (4 categories; 16.0%). Within Activities and Participation, categories were predominantly distributed in the d4 Mobility domain (14 categories; 66.7%), followed by d5 Self‐care (4; 19.0%), d6 Domestic Life (2; 9.5%), and d2 General Tasks and Demands (1; 4.8%). Secondary concepts were identified for seven items.

**Discussion:**

The resulting set of 25 ICF categories provides a structured description of the mobility and body function domains represented in the instrument. The application of ICF qualifiers to grade the severity of patient‐reported limitations represents a potential next step, subject to implementation and validation in applied clinical settings.

## 1 Introduction

Parkinson's disease (PD) is a neurodegenerative disorder with increasing prevalence and disability burden (GBD 2015 Neurological Disorders Collaborator Group 2017). Although not infectious, it has been described as a “Parkinson's pandemic” due to its projected global rise, with an estimated prevalence exceeding 17 million people by 2040 (Dorsey et al. 2018). Systematic documentation of health information in PD is essential for understanding its impact on functioning and identifying population needs (Cieza et al. 2019). When documented using a standardized language, such information enables comparability across services and countries, supporting more accurate estimates of needs and the development of targeted health policies and rehabilitation strategies (O'Young et al. 2019).

In this context, the World Health Organization developed the International Classification of Functioning, Disability and Health (ICF) as a standardized framework for describing health and disability within a biopsychosocial model (World Health Organization 2001). The ICF integrates body functions and structures, activities and participation, and contextual factors to describe functioning as the result of interactions between health conditions and environmental influences.

The ICF provides a structured system for describing functioning and is increasingly used in rehabilitation research and practice (Alford et al. 2015; Cieza et al. 2019). Given the complex clinical presentation of PD, characterized by heterogeneous motor and non‐motor manifestations, a biopsychosocial perspective is essential to capture the interaction between impairments, activity limitations, participation restrictions, and environmental factors (Raggi et al. 2011; van Uem et al. 2016). Although the use of the ICF has expanded internationally, its implementation in routine clinical practice remains limited (Madden and Bundy 2019; World Health Organization 2020).

One approach to operationalizing the ICF in practice is by linking existing assessment instruments to ICF categories using refined linking rules (Cieza et al. 2019). This method has been applied to different health measures, supporting their interpretation within the ICF (Carter et al. 2020; Pereira et al. 2022; Alflen et al. 2024). However, evidence remains limited for instruments embedded in clinical guidelines for people with Parkinson's disease (pwPD) (Vojciechowski et al. 2016).

To address this gap, the present study focused on selected components of the Pre‐Assessment Information Form (PIF), a pre‐assessment form recommended by the European Physiotherapy Guideline for Parkinson's disease (Keus et al. 2014). The PIF was designed to identify patient‐reported concerns prior to physiotherapy evaluation in people with Parkinson's disease. These components include selected items from the Patient Specific Index for Parkinson's Disease (PSI‐PD) (Nijkrake et al. 2009), which captures patient‐reported activity limitations, as well as the first question from the Questionnaire on the History of Falls (Stack and Ashburn 1999).

However, the PSI‐PD response format, based on a dichotomous classification of “difficult” or “not difficult,” provides limited detail regarding the specific domains of functioning represented by patients' reported difficulties. Linking these items to ICF categories may help clarify which aspects of functioning are represented within the instrument. In contrast to previous linkage studies involving broader health status or quality‐of‐life measures (Hagell et al. 2008), the present study focuses on a guideline‐recommended pre‐assessment form intended to identify patient‐reported concerns prior to physiotherapy evaluation in PD.

Therefore, this study aimed to link items from the PIF to ICF categories using the refined linking rules (Cieza et al. 2019), providing a structured description of the functioning domains represented in this instrument.

## 2 Materials and Methods

### 2.1 Study Design

This methodological study aimed to link items from the PIF to ICF categories using the refined linking rules proposed by Cieza et al. (2019).

### 2.2 Ethical Considerations

This study did not require ethics approval, as it did not involve human participants or identifiable personal data.

### 2.3 Instruments

#### 2.3.1 Pre‐Assessment Information Form (PIF)

The PIF, recommended by the European Physiotherapy Guideline for Parkinson's Disease (Keus et al. 2014), consists of the first two questions from the Questionnaire on the History of Falls (Stack and Ashburn 1999), one question from the New Freezing of Gait Questionnaire (NFOG‐Q) (Shine et al. 2012), the level of physical activity (Keus et al. 2014), and the first items of the Patient Specific Index for Parkinson's Disease (PSI‐PD) (Nijkrake et al. 2009), which lists 22 activities categorized under the domains of “walking,” “transfers,” and “manual activities”. The PIF also includes items addressing body functions, covering respiratory functions, muscle power, muscle tone, and pain.

#### 2.3.2 International Classification of Functioning, Disability and Health (ICF)

The ICF, developed by the WHO in 2001 (World Health Organization 2001), is a comprehensive classification system for health status, functioning, and disability at both individual and population levels. Its taxonomy is structured into two primary sections: Functioning and Disability, encompassing body functions (b), body structures (s), and activities and participation (d), and Contextual Factors, encompassing environmental factors (e) and personal factors (not classified within the ICF). Each component is represented by a letter followed by a numeric code, organized from chapter level (domain) (one digit) through second (two digits), third, and fourth levels (one digit each).

### 2.4 Linking Procedure

Cieza et al. (2002), (2005) proposed a method for linking existing health information to the ICF codes, which was further refined in 2019 (Cieza et al. 2019). Table 1 presents the 10 linking rules derived from this method:

**TABLE 1:** Specific ICF linking rules.

1.	Have a deep knowledge of the model and taxonomic bases of the ICF (domains and categories).
2.	Identify the most relevant concept to be related to the ICF.
3.	Identify, if necessary, the additional concepts contained in the item.
4.	Identify and document the perspective contained in the data collection when related to the ICF (capacity X performance).
5.	Identify and register the type of response category (frequency, intensity, duration…) and relate the concepts involved in answers as well.
6.	Relate all significant concepts (the most relevant) to the most precise ICF category.
7.	Use “other specified” (8) or “unspecified” (9) when appropriate.
8.	If the information provided by the concept involved in the items is not clear enough to identify the most precise ICF category, choose “not definable.”
9.	If a concept is not contained in the ICF but is clearly a personal factor, report it as such (pf).
10.	If a concept is not contained in any domain of the ICF, report it as “not covered” (nc).

*Note:* Adapted from Cieza et al. 2019.

Two evaluators (F.B.T.R. and G.S.P.), who possess extensive knowledge of the model and the ICF’s taxonomic foundations, independently performed the linking process. In cases of disagreement, a third evaluator (S.M.S.) was consulted to reach consensus. With more extensive ICF experience facilitated a consensus meeting and determined the final category assignment by applying the refined linking rules (Cieza et al. 2019).

The evaluators first identified the primary concept addressed in each activity and then linked the item to the relevant domains and categories within the ICF. Each concept was linked individually, allowing for the identification of additional concepts related to other domains in the ICF. In instances where the evaluators were unable to assign an item to a specific category, the classification “other specified” (Raggi et al. 2011) was utilized. The classification “unspecified” (Cieza et al. 2019) was used when the concept could be attributed to a particular domain but lacked sufficient information to assign it to a specific category within the ICF (Cieza et al. 2019).

When items contained more than one relevant concept, multiple ICF categories were assigned to distinguish between a primary category and additional categories. The primary category reflected the central construct of the item, while additional categories were used to capture secondary but relevant aspects.

### 2.5 Item Extraction

Eligibility criteria comprised items whose main or additional concepts corresponded to an ICF component of functioning and described or assessed the expression or extent of a problem. Furthermore, the perspective adopted in the items had to be descriptive (performance or capacity), indicating difficulty, or reflecting a need for assistance or dependency.

Exclusion criteria included items whose main or additional concepts were not aligned with the ICF framework, items adopting an appraisal perspective, and items with response options based on duration or qualitative attributions. These criteria were established to identify items with the potential to inform the development of a functioning metric and relevant determinants of functioning in the older adult population (Moreira et al. 2024).

### 2.6 Data Analysis

To assess the level of agreement between the evaluators regarding the linkage of each item, Kappa coefficients (*k*) with a 95% confidence interval (CI) were calculated. The interpretation of the *k* values was as follows: 0.00 = no agreement; 0.01 to 0.20 = insignificant agreement; 0.21 to 0.40 = low agreement; 0.41 to 0.60 = moderate agreement; 0.61 to 0.80 = good agreement; and 0.81 to 1.00 = almost perfect agreement (Landis and Koch 1977). IBM's Statistics for Windows (version 22.0, IBM Corp., Armonk, NY, USA) was employed for all statistical analyses, with a significance level of 5% (*p* < 0.05).

## 3 Results

Initial agreement between the two independent evaluators was moderate (*k* = 0.42–0.54; *p* < 0.001). Agreement decreased as the specificity of the ICF taxonomy level increased, reflecting the greater interpretive complexity of finer categorical distinctions (Table 2). Disagreements between the two evaluators were of two distinct types. The first, and more frequent, involved taxonomy‐level discrepancies: both evaluators identified the same conceptual domain but assigned the item to different levels of the ICF hierarchy (e.g., second vs. third level). The second type, less frequent, involved conceptual discrepancies: divergence in identifying the primary construct underlying the item. A consensus meeting was held, facilitated by a third evaluator (S.M.S) with more extensive ICF experience, who reviewed each discordant item, considered the documented justifications provided by both evaluators, and applied the refined linking rules to determine the final category assignment. In all cases, the most specific ICF category supported by the item's primary construct was selected, consistent with the linking rule. Disagreements were most frequent at the third taxonomy level and concentrated in items describing gait under specific conditions—notably start walking, stop walking, and walking through narrow passages—where evaluators diverged between d450 Walking and more specific subcategories (d4508 Walking, other specified; d4503 Walking around obstacles).

**TABLE 2:** Inter‐rater agreement on linking of pre‐assessment information form (PIF) items to the international classification of functioning, disability and health (ICF).

ICF taxonomy level	Kappa coefficient (95% CI)	Interpretation
First level category	0.54 (0.49–1.00)^*^	Moderate agreement
Second level category	0.53 (0.36–0.83)^*^	Moderate agreement
Third level category	0.42 (0.12–0.77)^*^	Moderate agreement

*Note:* Kappa (*k*) coefficients and 95% confidence intervals for ICF domains and first, second and third category levels.

^*^ *p* < 0.001 for all kappa measurements and 95% confidence interval.

A total of 26 items from the PIF were linked to the ICF, resulting in 34 linked concepts mapped to 25 unique ICF categories. Most linked concepts were assigned to the Activities and Participation component (21 categories; 84.0%), while a smaller proportion was linked to Body Functions (4 categories; 16.0%). Within Activities and Participation, categories were predominantly concentrated in the mobility domain (d4; *n* = 14; 66.7%), followed by self‐care (d5; *n* = 4; 19.0%), domestic life (d6; *n* = 2; 9.5%), and general tasks and demands (d2; *n* = 1; 4.8%) (Table 3).

**TABLE 3:** Linking of pre‐assessment information form (PIF) items to the international classification of functioning, disability and health (ICF).

PIF item	ICF domains	Main ICF category	Additional ICF category
Walking indoors	d4 mobility	d450 walking	—
Walking outdoors	d4 mobility	d450 walking	—
Turning	d4 mobility	d410 changing basic body position	—
Start walking	d4 mobility	d4508 walking, other specified	—
Climbing and descending stairs	d4 mobility	d4551 climbing	—
Walking while performing dual tasks	d4 mobility	d450 walking	d2200 carrying out multiple tasks
Walking through narrow passages	d4 mobility	d4503 walking around obstacles	—
Stop walking	d4 mobility	d4508 walking, other specified	—
Turning in bed	d4 mobility	d4107 rolling over	—
Getting into or out of bed	d4 mobility	d4100 lying down	d4104 standing
Getting into or out of a car	d4 mobility	d470 using transportation	—
Getting into or out of a chair	d4 mobility	d4103 sitting	d4104 standing
Getting onto or off a toilet seat	d4 mobility	d4103 sitting	d4104 standing
Getting into or out of a bath	d4 mobility	d4109 changing basic body position, unspecified	—
Picking up an object from the floor	d4 mobility	d4400 picking up	d4101 squatting
Getting up from the floor	d4 mobility	d4104 standing	—
Getting on or off a bicycle	d4 mobility	d4750 driving human‐powered transportation	d4103 sitting/d4104 standing
Preparing meals	d6 domestic life	d630 preparing meals	—
Doing housework	d6 domestic life	d640 doing housework	—
Eating	d5 self‐care	d550 eating	d560 drinking
Washing	d5 self‐care	d510 washing oneself	—
Dressing	d5 self‐care	d540 dressing	—
Easily out of breath	b4 functions of the cardiovascular, hematological, immunological and respiratory systems	b440 respiratory functions	—
Muscle weakness	b7 neuromusculoskeletal and movement‐related functions	b730 muscle power	—
Stiffness	b7 NEUROMUSCULOSKELETAL and movement‐related functions	b735 muscle tone functions	—
Pain	b2 sensory functions and pain	b280 sensation of pain	—

*Note:* All items were assessed from the perspective of functional capacity. Response options for PSI‐PD items: *Difficult*/*Not difficult*. Classification refers to ICF linking rules outcomes: *Other specified* = concept identifiable within a domain but not fitting an existing specific category; *Unspecified* = concept attributable to a domain but lacking sufficient detail for a specific category assignment.

Abbreviations: PIF, pre‐assessment information form; PSI‐PD, patient specific index for Parkinson's disease.

The category d450 Walking was the most frequently assigned, encompassing items describing gait performance under different conditions. Although environmental context may influence walking performance, items referring to indoor and outdoor walking were linked to the same ICF category (d450 Walking), as both share the same primary meaningful concept. In addition, the ICF does not provide distinct categories to differentiate walking according to environmental context, and therefore the closest matching category was selected according to the linking rules proposed.

Two items—start walking and stop walking—were linked to d4508 Walking, other specified, as neither gait initiation nor gait termination corresponds to an existing specific ICF third‐level category. Items reflecting multidimensional activities were assigned multiple categories when the item content clearly involved more than one distinct ICF construct. For example, walking while performing dual tasks was linked to both d450 Walking and d2200 Carrying out multiple tasks, reflecting the motor and cognitive‐executive demands commonly impaired in PD. To increase transparency of the linkage process, representative examples illustrating the rationale underlying category assignment decisions are provided in Table S1.

The terms not covered (nc) and personal factor (pf) were not required for any item, indicating that all PIF content fell within the ICF framework. The linkage also revealed that the PIF does not generate linked concepts from the ICF domains of Interpersonal Interactions and Relationships (d7), Major Life Areas (d8), Community, Social and Civic Life (d9), or Environmental Factors (component e).

## 4 Discussion

Linking PIF items to the ICF allows patient‐reported difficulties to be organized into ICF categories relevant to physiotherapy assessment in pwPD. As shown in Table 3, the identified categories were predominantly distributed within the Activities and Participation component, with a clear concentration in the d4 Mobility domain. Four body function categories were also identified, capturing symptom‐related aspects that complement the activity‐based content of the instrument.

Some activities involved more than one meaningful concept. For example, dual‐task walking and postural transitions combine mobility and cognitive demands, requiring multiple ICF category assignments. These findings reflect the emphasis of the PIF on walking activities and postural transitions, which are commonly affected in pwPD and frequently addressed in physiotherapy practice. The frequent linkage to categories such as d450 Walking and d410 Changing basic body position further supports the alignment between the content of the PIF and functional domains commonly assessed in physiotherapy. At the same time, the findings underscore important limitations of the current ICF taxonomy in representing specific motor control impairments associated with gait dysfunction in PD. Clinically relevant manifestations such as festination and gait initiation hesitation were not sufficiently differentiated within the available ICF categories, requiring linkage to broader or conceptually related classifications that did not fully capture their specific motor characteristics.

The moderate inter‐rater agreement reflects the conceptual overlap and hierarchical complexity of the ICF taxonomy, which may generate divergent categorizations even among evaluators with equivalent expertise during independent, blinded linking. Initial disagreements were predominantly of two types: taxonomy‐level discrepancies, in which both evaluators identified the same conceptual domain but differed in the hierarchy level assigned, and conceptual discrepancies, involving divergence in the identification of the primary construct. These were resolved through a structured consensus process facilitated by a third evaluator to determine the most specific category supported by each item's content (Cieza et al. 2019). This level of agreement is consistent with published ICF linkage studies: Pereira et al. (2022) reported kappa values of 0.48–0.73 across taxonomy levels when linking post‐stroke instruments, and Alflen et al. (2024) obtained *k* = 0.52 when linking an environmental quality measure. A decrease in agreement with increasing category specificity is expected and has been consistently reported in the ICF linkage literature.

To our knowledge, this is the first study to link the PIF to the ICF using the refined linking rules. This contrasts with the only previous instrument‐level linkage study in PD, which focused on the PDQ‐39 as a quality‐of‐life measure (Hagell et al. 2008). This distinction is relevant because the PIF was developed to identify patient‐reported difficulties prior to physiotherapy evaluation within the European Physiotherapy Guideline for PD. In addition, the refined linking rules incorporate updated recommendations regarding perspective documentation and response option classification that were not included in earlier versions of the methodology.

Another relevant implication is the potential to improve the granularity of functional assessment. Although the PIF uses dichotomous response options, linking items to ICF categories allows the use of qualifiers to describe the severity of limitations in a more standardized manner. This may provide a conceptual basis for future studies exploring the application of ICF qualifiers to describe the severity of patient‐reported functional limitations. However, these potential applications require further implementation and validation studies.

The distribution of ICF categories observed in this study is consistent with previous findings using ICF checklists in PD, which also identified mobility as the most affected domain, followed by self‐care and domestic life (Raggi et al. 2011). Categories from the mobility domain predominated (66.7%), followed by self‐care (19.0%), domestic life (9.5%), and general tasks and demands (4.8%), reflecting a comparable pattern of functional limitation. Mobility limitations are clinically relevant in PD because of their association with adverse outcomes such as falls, loss of independence, institutionalization, and reduced quality of life (Bouça‐Machado et al. 2018; Hsu et al. 2018). Previous studies have also explored the relationship between ICF‐based functional classification and functional outcomes in PD, including walking ability and disease severity (Chiu et al. 2013; Teng et al. 2013; Hsu et al. 2018). In this context, the predominance of categories from the mobility domain within the PIF further supports its alignment with domains commonly addressed during physiotherapeutic assessment in pwPD.

Although categories from the mobility domain predominated, the linkage process also highlighted important domains insufficiently represented within the PIF relative to the broader biopsychosocial profile of PD. Participation restrictions represent a domain of particular clinical relevance that is not captured by the PIF. A systematic review by Kim et al. (2024) examined the conceptualization and measurement of participation in PD across 36 studies and found that fewer than half explicitly defined participation, and that 18 different instruments have been used for this purpose without methodological consensus. This fragmentation illustrates that participation in PD remains both clinically significant and systematically underassessed. Restrictions across social roles, employment and economic life, and community and civic activities are well‐documented consequences of PD with direct implications for quality of life and caregiver burden (van Uem et al. 2016), yet fall outside the scope of PIF items. Cognitive functions, including attention, executive function, and working memory—increasingly targeted in dual‐task and fall prevention physiotherapy in PD—are also absent, as are communication functions and environmental factors. The absence of these domains does not constitute a flaw in the PIF, which was designed for pre‐assessment triage rather than comprehensive functioning classification. However, it does identify the need for complementary instruments when a full ICF‐based functional profile is required in PD rehabilitation.

Although the findings are relevant, this study has some limitations that should be considered when interpreting the findings. First, the linking process was based on a specific set of items from the PIF, and therefore the results reflect only the content of these selected instruments rather than the full spectrum of patient‐reported functioning in Parkinson's disease. Second, the use of a third evaluator to resolve disagreements, while consistent with established ICF linkage procedures, may introduce interpretive influence on final category assignments that cannot be fully quantified; the moderate kappa values indicate that classification at finer taxonomy levels retains an element of judgment even among trained evaluators. Finally, the practical applicability of the proposed linkage remains to be evaluated in applied settings, including its feasibility for routine physiotherapy documentation and its acceptability among physiotherapists with different levels of ICF familiarity. Future studies should investigate whether ICF‐coded PIF data can be integrated into existing documentation systems and used in clinical settings.

### 4.1 Implications for Physiotherapy Practice

Linking PIF items to the ICF provides a structured description of the functioning domains represented in this guideline‐recommended pre‐assessment form, particularly those related to mobility and movement‐related body functions. The predominance of categories related to mobility, self‐care, and domestic life reflects domains commonly addressed during physiotherapy assessment in PD. In addition, the linkage process identified domains that are not represented within the PIF, including participation, cognition, and environmental factors, which may require complementary assessment when broader characterization of functioning is needed. Although future application of ICF qualifiers may be explored, implementation feasibility and clinical utility were not evaluated in the present study.

## Author Contributions

**Fernanda Botta Tarallo Rogatto:** conceptualization, data curation, formal analysis, investigation, methodology, writing – original draft. **Érica Tardelli Neves Guelfi:** supervision, writing – original draft, writing – review and editing. **Vívian Elaine Vargas Alflen:** writing – review and editing. **Leandro Bruno Barbosa Silva:** writing – review and editing. **Gabriela Santos Pereira:** data curation, formal analysis, writing – review and editing. **Soraia Micaela Silva:** conceptualization, data curation, formal analysis, investigation, methodology, project administration, supervision, writing – original draft, writing – review and editing.

## Funding

The authors have nothing to report.

## Ethics Statement

The authors have nothing to report.

## Consent

The authors have nothing to report.

## Conflicts of Interest

The authors declare no conflicts of interest.

## Supporting information


**Table S1:** d450 Walking, d2200 Carrying out multiple tasks, d4103 *Sitting*, d4104 Standing.

## Data Availability

The data that support the findings of this study are available from the corresponding author upon reasonable request.
