# Supplementary material for: Linking the Pre‐Assessment Information Form (PIF) to the ICF: Enhancing Standardized Functional Assessment in Parkinson's Disease
Source: Physiother Res Int. 2026 Jun 13;31(3):e70247. doi: 10.1002/pri.70247 (PMC13264484; doi:10.1002/pri.70247)
Supplement: Supplementary file 1 — Table S1: d450 Walking, d2200 Carrying out multiple tasks, d4103 Sitting, d4104 Standing. [file PRI-31-e70247-s001.docx]

**S1.** Supplemental File.

| **PIF item** | **Meaningful concept** | **Linking rationale** | **Final ICF category** |
| --- | --- | --- | --- |
| Start walking | Gait initiation | No sufficiently specific ICF category exists for gait initiation | d4508 Walking, other specified |
| Walking while performing dual tasks | Walking + simultaneous task execution | Item contains two meaningful concepts | d450 + d2200 |
| Getting into or out of a chair | Sitting-to-standing transition | Item involves sequential postural transitions | d4103 + d4104 |

| **Caption:** d450 Walking, d2200 Carrying out multiple tasks, d4103 Sitting, d4104 Standing. |
| --- |
